# Ni-Doping as a Design Strategy for Fe-Based Catalysts: Towards Efficient CO_2_ Activation and Dissociation Studied by Density Functional Theory

**DOI:** 10.3390/nano16181165

**Published:** 2026-09-16

**Authors:** Ana Cecilia Rossi Fernández, Silvia Andrea Fuente, Ricardo Mario Ferullo, Alfredo Juan, Patricia Gabriela Belelli

**Affiliations:** 1 Instituto de Física del Sur (IFISUR), Universidad Nacional del Sur (UNS), CONICET, Av. L.N. Alem 1253, Bahía Blanca B8000CPB, Argentina; anarossi@ifisur-conicet.gob.ar (A.C.R.F.); silviafuente@frbb.utn.edu.ar (S.A.F.); 2 Instituto de Química del Sur (INQUISUR), Universidad Nacional del Sur (UNS), CONICET, Av. L.N. Alem 1253, Bahía Blanca B8000CPB, Argentina; caferull@criba.edu.ar

**Keywords:** CO_2_ activation, Fe-Ni bimetallic catalysts, Density Functional Theory (DFT), CO_2_ dissociation, spin-resolved d-band analysis

## Abstract

In this work, we investigate the activation of CO_2_ on bimetallic Ni–Fe catalysts, using Fe as the base metal and Ni as a dopant at low concentrations. Our aim is to evaluate how the presence of Ni, either as a substitutional atom or as an adatom, influences CO_2_ activation and facilitates subsequent C–O bond breaking. The catalytic surfaces were modeled using Fe(100) slabs, and the effect of Ni was examined in three configurations: (I) substitution of one Fe atom by Ni in the first layer, (II) Ni as an adatom, and (III) Fe as an adatom on the Ni-substituted Fe surface. In all cases, the optimized geometries lead to CO_2_ activation. The corresponding energy profiles for C–O bond dissociation were obtained and compared with those on pure Fe(100) (E_act_ = 0.84 eV). Among the three systems, the configuration with substitution of Fe by Ni in the first layer exhibits the lowest activation barrier for C–O bond breaking, just 0.48 eV—a substantial reduction of 0.36 eV relative to pristine Fe(100). Only the Ni_ad_-Fe(100) surface yields a higher barrier (0.93 eV). These results are rationalized by extracting the spin splitting from the spin-resolved LDOS of the metal d-band centers, revealing that the superior performance of Ni_1_-Fe(100) arises from a more efficient local spin reorganization during bond dissociation.

## 1. Introduction

The rapid growth of the global economy and continued dependence on fossil fuels have led to a sharp increase in atmospheric CO_2_ levels, intensifying the greenhouse effect and contributing to more frequent extreme weather events. Consequently, mitigating CO_2_ emissions has become a global priority. At the same time, CO_2_ serves as an abundant, inexpensive, and non-toxic carbon source. From the perspective of green chemistry and sustainable development, its conversion into value-added chemicals represents an attractive strategy to simultaneously reduce emissions and alleviate carbon-resource limitations [1].

Conventional carbon capture and utilization (CCU) technologies involve multiple stages—including CO_2_ adsorption, adsorbent regeneration, compression, and transportation—which reduce overall energy efficiency and introduce challenges such as material degradation. To address these limitations, the Integrated CO_2_ Capture and Utilization (ICCU) process has emerged as a promising alternative, enabling the direct conversion of captured CO_2_ into valuable products within a single reactor [2]. Therefore, the development of dual-functional materials (DFMs) that combine adsorption and catalytic sites within a single system is essential.

A major challenge in CO_2_ conversion is the activation of the molecule itself. The high thermodynamic stability of the C=O bonds and the multi-electron nature of CO_2_ reduction often lead to low activity, poor selectivity, and catalyst deactivation in processes such as methanation, the reverse water-gas shift (RWGS), and dry reforming of methane (DRM). Although numerous transition-metal catalysts have been explored for CO_2_ hydrogenation [3,4], the rate-limiting step often remains the activation and cleavage of the C–O bond. A molecular-level understanding of the interaction between CO_2_ and metal surfaces is therefore crucial for designing more efficient catalysts [5,6].

CO_2_ activation typically involves charge transfer from the metal surface to the antibonding orbitals of the molecule, inducing a bent CO_2_^δ−^ geometry that acts as a precursor to C–O bond dissociation [7]. Thus, elucidating the adsorption and stabilization of this CO_2_^δ−^ species is fundamental. Wang et al. [8] investigated CO_2_ chemisorption across a series of transition metals (Fe, Co, Ni, Cu, Rh, Pd, Ag, Pt, Au), demonstrating that the adsorption strength and activation correlate with the d-band center position and the magnitude of charge transfer. Motivated by these insights, our work focuses on bimetallic Ni–Fe systems and investigates how their tailored electronic structure can enhance CO_2_ activation.

Nickel and iron are compelling candidates for several reasons. First, both metals are well-known active centers for CO_2_ reduction; a biological example is the Ni–Fe active site in carbon monoxide dehydrogenase, which catalyzes CO_2_-to-CO conversion under mild conditions [9,10]. Second, Fe is inexpensive, and its surface properties can be tuned for catalytic and electrochemical applications. Ko et al. [7] reported that alloys featuring Fe, Ru, Co, and Ni as host metals strongly activate CO_2_, exhibiting low dissociation barriers and favorable segregation behavior. Among these candidates, the Fe–Ni combination offers a favorable cost–performance balance. On the other hand, Ni-based systems supported on SiO_2_ and Al_2_O_3_ are widely studied for DRM [11,12,13]. It has been shown that the selectivity toward C_2_^+^ hydrocarbons is enhanced by adding a second metal (Co, Ni, Cu, Pd) into Fe-based catalysts supported on Al_2_O_3_ [14,15,16]. In particular, Ni incorporation into Fe increases synthetic natural gas production and improves the activity of the reverse water-gas shift reaction [17,18]. In addition, our previous theoretical study on Ni-doped Fe(111) surfaces has shown that Fe–Ni alloys can strongly activate CO_2_ [19]. These features make the Ni-doped Fe(100) surface a suitable model system for investigating how the incorporation of Ni as a second metal modifies the reactivity of Fe toward CO_2_.

Surface morphology also plays a critical role in catalytic performance. CO_2_ dissociation is generally unfavorable on flat terraces but is significantly promoted by topological defects such as steps, kinks, and corners [20]. However, DFT studies addressing CO_2_ activation on defective transition-metal surfaces remain limited, with most research focusing on Cu [21,22]. Metal adatoms can strongly modify adsorption processes by altering the local electronic structure, adsorption geometry, bond strength, and work function [23,24]. Our recent theoretical research showed that specific topological defects, such as adatoms and edge sites (represented by the Fe(310) zigzag surface formed by the intersection of the (100) and (110) planes), play an important role in CO_2_ dissociation. The activation barrier decreases by approximately 20–25% relative to Fe(100) [25]. Doping transition-metal surfaces—including oxides, pure metals, and bimetallic systems—represents a well-established strategy for tailoring catalytic, magnetic, optical, and electronic properties [26,27]. Among these strategies, isolating the dopant as a single atom—an approach commonly referred to as a Single-Atom Alloy (SAA)—has attracted growing interest, as it allows the intrinsic electronic effect of the dopant to be decoupled from ensemble effects that typically arise at higher dopant concentrations [28,29]. Comparing this isolated configuration with a dopant adatom (point defect) further enables a systematic assessment of how each configuration individually influences CO_2_ activation, an aspect that remains largely unexplored in the literature. To our knowledge, no previous study has directly compared these two dopant configurations—isolated substitutional atom versus adatom—for CO_2_ activation on the same host surface.

In this context, we investigate the influence of Ni doping and the presence of Fe or Ni adatoms on CO_2_ activation on the Fe(100) surface. Three surface configurations were evaluated using density functional theory (DFT) as implemented in the VASP code: (i) substitution of one Fe atom by Ni in the first surface layer, (ii) a Ni adatom on pure Fe(100), and (iii) an Fe adatom on the Ni-substituted Fe(100) surface. In all cases, CO_2_ adsorption leads to molecular activation. The Fe(100) surface was selected as the reference substrate because its relatively simple and well-defined structure provides a suitable model for isolating the effects of Ni incorporation and adatom formation. Fe(100) has also been extensively investigated in our previous theoretical studies of CO_2_ adsorption and dissociation [25], which serve as a well-established baseline for the present analysis. Furthermore, the present work builds upon our previous study of CO_2_ dissociation on Ni-doped Fe(111) [19], extending the investigation to the Fe(100) surface and allowing us to examine how the local atomic configuration and surface structure influence CO_2_ activation.

## 2. Computational Details

All calculations were performed using the Vienna ab initio Simulation Package (VASP) [30] within the framework of density functional theory (DFT) [31]. The projector augmented-wave (PAW) method [32,33] and the generalized gradient approximation (GGA) with the Perdew–Burke–Ernzerhof (PBE) functional [34] were employed to describe the exchange–correlation energy. A plane-wave cutoff energy of 400 eV was used in all calculations. Spin polarization was included to properly account for the magnetic nature of the systems. Brillouin-zone integrations were carried out using a 3 × 3 × 1 Monkhorst-Pack k-point mesh. Structural optimizations were considered converged when the atomic forces were below 0.01 eV/Å. The pure Fe(100) surface used as the substrate for the Ni-doped systems, and taken as the reference for comparison throughout this work, corresponds to our previous work [25]. The computational parameters employed therein remain fully consistent with those applied in the present study. Given this methodological consistency, the adsorption energy (E_ads_) and activation energy (E_act_) values reported below for the Ni-doped systems are directly comparable with those previously obtained for pure Fe(100).

Three Fe(100)-based surfaces were modeled:

- Ni_1_-Fe(100), represented by substituting a single Fe atom with Ni in the first surface layer;
- Ni_ad_-Fe(100), created by adding a Ni adatom on Fe(100);
- Fe_ad_-Ni_1_-Fe(100), generated by adding an Fe adatom on the Ni-substituted Fe(100) surface through the formation of a Ni-Fe_ad_ bond.

**Figure 1:**
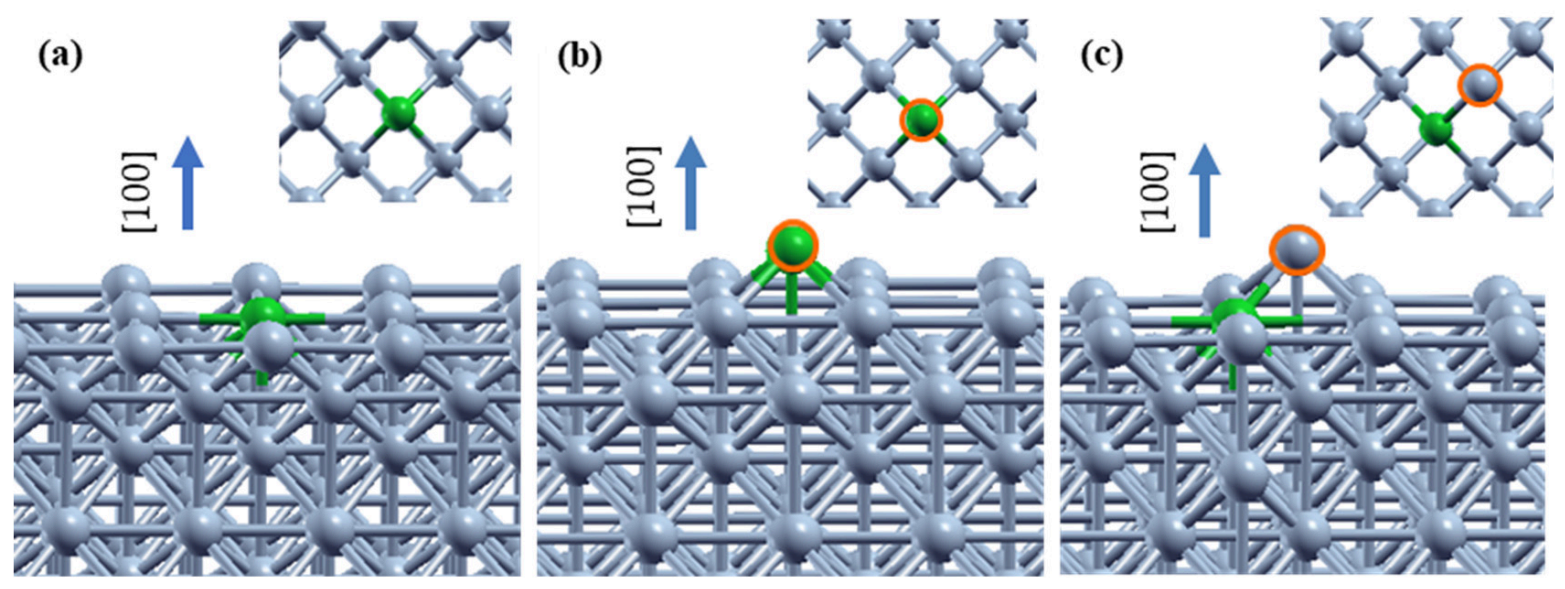
Side and top views of the three surfaces modeled: (**a**) Ni_1_-Fe(100), (**b**) Ni_ad_-Fe(100), and (**c**) Fe_ad_-Ni_1_-Fe(100). Color code: Fe (light grey) and Ni (green). The Ni and Fe adatoms are highlighted with orange circles.

All surfaces were modeled using a periodic *p*(3 × 3) six-layer slab. The systems comprised: 53 Fe + 1 Ni (Ni_1_-Fe), 54 Fe + 1 Ni adatom (Ni_ad_-Fe), and 53 Fe + 1 Ni + 1 Fe adatom (Fe_ad_-Ni_1_-Fe). The Ni dopant coverage is 1/9. Figure 1 shows the three surface models. During geometry optimization, the three topmost layers and all adsorbates were allowed to relax, while the remaining layers were fixed at their calculated bulk positions. A vacuum gap of 12 Å was included to avoid spurious interactions between periodic images. The slabs were constructed using the optimized lattice parameter of bulk BCC Fe (2.833 Å), which agrees well with previous theoretical and experimental values [35,36,37,38,39,40]. The calculated magnetic moment of 2.2 μB per Fe atom is consistent with literature data [35,37,38,41].

The stabilities of the bimetallic surfaces were compared with respect to that of pure Fe(100) (E_Fe(100)_). For this purpose, formation energies (E_form_) were calculated relative to the bulk energies of Ni (E_Ni_) and Fe (E_Fe_):

E_form_ = E_surf_ − E_Fe(100)_ − E_Ni_ + *n* ∙ E_Fe_

where E_surf_ corresponds to the energy of Ni_1_-Fe(100) or Ni_ad_-Fe(100), and the parameter *n* takes values of 0 and 1 for Ni_ad_-Fe(100) and Ni_1_-Fe(100), respectively.

The results indicate a formation energy of −0.30 eV for the substitution of one Fe atom by Ni on the Fe(100) surface (Ni_1_-Fe(100)), whereas the formation of the Ni adatom yielded +0.24 eV (Ni_ad_-Fe(100)). Comparing these energetics indicates that the substitutional dopant is thermodynamically more stable than the adatom configuration. The Ni_ad_-Fe(100) and Fe_ad_-Ni_1_-Fe(100) surfaces contain the same number of atoms but differ in their atomic distribution, and the energy difference between them is 0.13 eV, suggesting a low interconversion cost.

Transition states were identified using the climbing-image nudged elastic band (CI-NEB) method [42], and each transition state was subsequently verified by vibrational analysis to ensure the presence of a single imaginary frequency along the reaction coordinate.

The adsorption energy (E_ads_) was computed as:

E_ads_ = E_mol/surf_ − E_surf_ − E_mol_ (1)

where E_mol/surf_ represents the total energy of the adsorbed molecules/species with catalyst surface, E_surf_ is the energy of the bare surface, and E_mol_ is the energy of the molecules/species in the gas phase.

Reaction energies (E_reac_) for dissociation or formation processes were calculated as:

E_reac_ = E_FS_ – E_IS_ (2)

where E_IS_ and E_FS_ are the total energies of the initial state and final state, respectively.

Activation energies (E_act_) were obtained from:

E_act_ = E_TS_ − E_IS_ (3)

where E_TS_ is the energy of the transition state. A more negative E_ads_ indicates stronger adsorption, and negative E_reac_ values correspond to exothermic reactions. All energy values reported in this study include zero point-energy (ZPE). For comparison, the complete set of energy values with and without ZPE corrections is presented in Tables S1–S4 of the Supplemental Materials.

Net atomic charges (*q*) and charge transfer were evaluated using the DDEC6 (Density Derived Electrostatic and Chemical) partitioning method. In this approach, net atomic charges are calculated using the Chargemol software (Version 3.4.4) [43], which decomposes the total electron density obtained from VASP into individual atomic contributions. This method simultaneously optimizes electrostatic potential fitting and chemical state expectations for each atom. Additionally, the electronic charge-density difference (∆*ρ*) was also calculated to investigate the electronic charge reorganization due to the adsorption process, according to the following expression:

∆*ρ* = *ρ*_CO2/surf_ − *ρ*_surf_ − *ρ*_CO2_ (4)

where *ρ*_CO2/surf_ is the charge density of the CO_2_ molecule interacting with the corresponding surface. *ρ*_surf_ is the charge density of the surface and *ρ*_CO2_ is the charge density of the CO_2_ molecule, with both densities calculated as isolated fragments but in the corresponding geometry after the adsorption process.

Within the DDEC6 framework [44], the bond order provides a quantitative measure of the electron density shared between two atoms, derived directly from the DFT electron density. Variations in the DDEC6 bond order along the reaction pathway offer a clear electronic signature of bond weakening and formation, allowing a direct correlation with CO_2_ activation and dissociation barriers on the Ni–Fe bimetallic surfaces. Therefore, in this work, bond-order analysis was performed for selected systems.

We analyzed the local density of states (LDOS) for the three pure Ni-Fe surfaces, as well as for initial states with CO_2_ adsorbed on each surface and their corresponding transition states. To this end, a refined 7 × 7 × 1 Monkhorst-Pack *k*-point mesh was employed for electronic structure property calculations and DOS integration to guarantee accurate spin-resolved results. To gain deeper insight into surface reactivity and its correlation with activation barriers, we calculated the overall d-band center (E_d_), the d-band centers for the spin-up (E_↑_) and spin-down (E_↓_) channels, and the spin splitting (ΔE = E_↑_ − E_↓_) for selected LDOS profiles.

## 3. Results and Discussion

### 3.1. CO_2_ Adsorption and Activation on Ni_1_-Fe(100), Ni_ad_-Fe(100), and Fe_ad_-Ni_1_-Fe(100)

Upon chemisorption, the CO_2_ molecule undergoes significant structural deformation, characterized by a deviation from linearity and elongation of the C–O bonds. These geometric changes are clear indicators of molecular activation, driven by charge transfer from the metal surface to antibonding orbitals. To evaluate CO_2_ activation on the three surfaces, we systematically searched for adsorption configurations associated with the specific sites present on each surface.

#### 3.1.1. CO_2_ Adsorption on Ni_1_-Fe(100)

**Figure 2:**
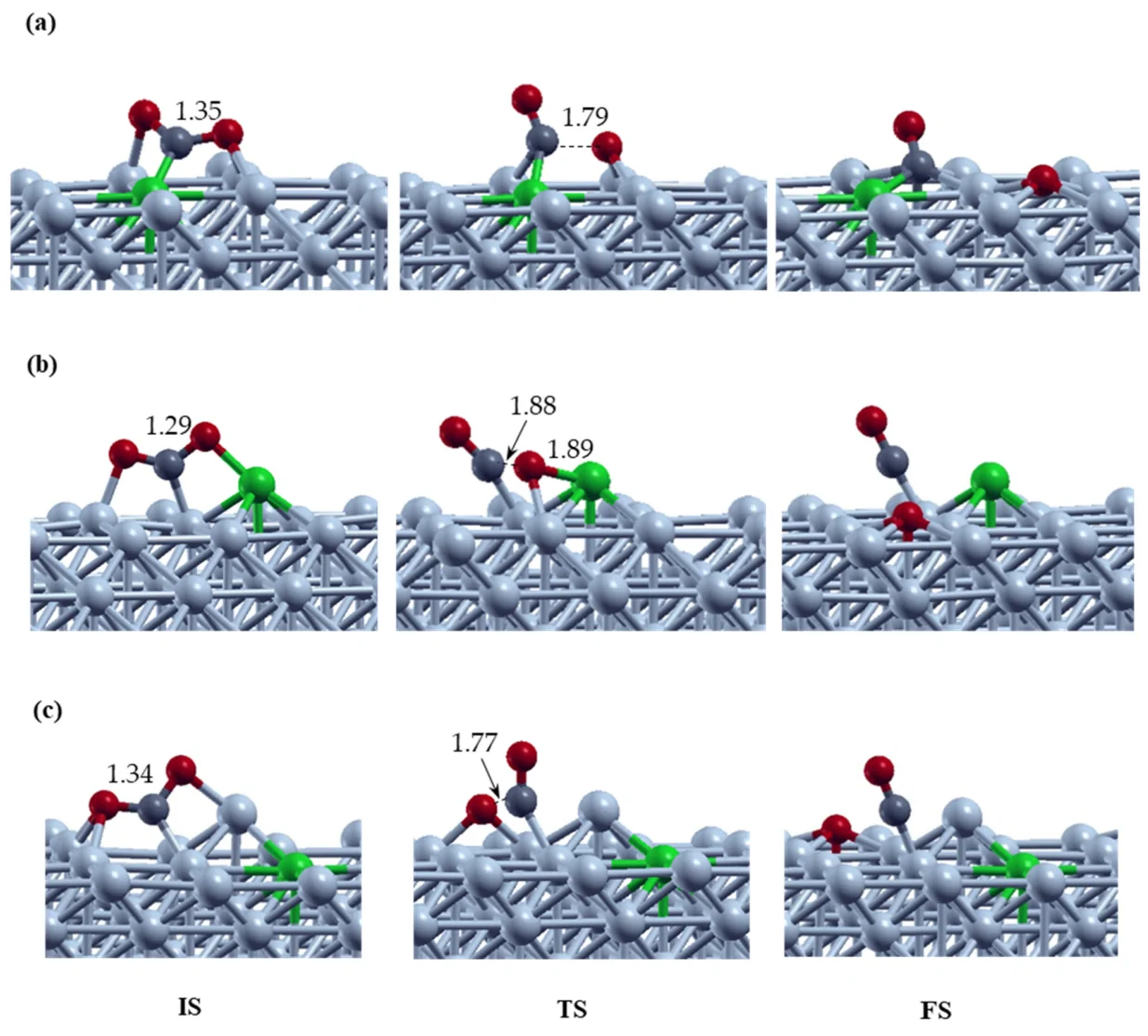
Adsorption geometries of initial (IS), transition (TS) and final states (FS) of CO_2_ dissociation on: (**a**) Ni_1_-Fe(100)-IA, (**b**) Ni_ad_-Fe(100)-IIA, and (**c**) Fe_ad_-Ni_1_-Fe(100)-IIIA. Color code: Fe (light grey), Ni (green), C (dark grey), and O (red). Distances in Angstroms (Å).

Four adsorption modes (IA–ID) were identified (Figure 2a and Figure S1). The three hollow-site configurations (IA–IC) represent the most stable states, with adsorption energies ranging from −0.74 to −0.96 eV (Table 1). These results are consistent with the findings of Nie et al. [45], who also reported hollow sites as the preferred adsorption geometry on Fe–Ni surfaces. Notably, the IA–IC modes also show the highest degree of molecular activation, with one C–O bond elongating to 1.34–1.35 Å, compared to the gas-phase value of 1.18 Å. In contrast, mode ID, where CO_2_ adopts a bridge geometry, is less stable and exhibits significantly lower activation. In the most stable and activated configuration (IA), the carbon atom lies closer to the Ni dopant (1.92 Å; Figure 2a and Table 2). In IB and IC, the carbon atom interacts more strongly with a neighboring Fe atom (1.97 Å), with the Ni atom playing a secondary role.

DDEC6 charge analysis reveals substantial charge transfer from the surface to the CO_2_ molecule, forming a negatively charged CO_2_^δ–^ species. In agreement with Nie et al. [45], we found that stronger adsorption correlates with more pronounced charge transfer. Thus, the IA–IC modes exhibit greater charge accumulation on CO_2_ compared to the ID configuration (Table 1).

#### 3.1.2. CO_2_ Adsorption on Ni_ad_-Fe(100)

Three adsorption modes (IIA–IIC) were identified on this surface (Figure 2b and Figure S2). Configurations IIA and IIB correspond to bridge geometries near the Ni adatom (Table 3). Among these, IIA is both the most stable and the most activated. The carbon atom interacts with the nearest Fe atom, while one oxygen atom binds to the Ni adatom at 1.94 Å (Figure 2b and Table 2). In contrast, IIB is considerably less stable and exhibits lower activation: although the carbon atom interacts more closely with the Ni adatom (1.90 Å), the activated C–O bond elongates only to 1.26 Å (Figure S2). Despite being the least stable configuration, mode IIC is noteworthy because CO_2_ adsorbs directly atop the Ni adatom without interacting with Fe atoms. The C–Ni distance is 1.94 Å (Figure S2).

Charge transfer to CO_2_ is observed in all cases and correlates with adsorption stability, with more stable systems exhibiting more pronounced charge transfer. However, the overall charge transfer is lower than that observed for the Ni_1_-Fe(100) system, suggesting that substitutional Ni is more effective at promoting CO_2_ activation than an isolated adatom.

#### 3.1.3. CO_2_ Adsorption on Fe_ad_-Ni_1_-Fe(100)

This surface exhibits the largest number of adsorption modes (IIIA–IIIE), reflecting the increased variety of geometric environments created by the Fe adatom (Figure 2c and Figure S3). Configuration IIIA is the most stable, with CO_2_ adsorbing on a hollow site while interacting simultaneously with both the Fe adatom and the Fe surface (Table 4). Notably, it does not interact with the Ni dopant due to spatial separation. This system exhibits stronger adsorption than Ni_ad_-Fe(100), even though both contain the same number of atoms. The carbon atom interacts with a surface Fe atom at 1.96 Å, while one oxygen atom binds to the Fe adatom at 2.00 Å (Figure 2c and Table 2). The highest activation (C–O = 1.34 Å) occurs when the activated oxygen coordinates with a surface Fe atom (2.05 Å).

The IIIB configuration is the second most stable, with an adsorption energy equivalent to that on Fe(100) [25]. CO_2_ remains bound to the Ni dopant (C–Ni = 1.91 Å), while one oxygen binds to the Fe adatom (1.98 Å) (Figure S3). The second oxygen also interacts with a surface Fe atom (2.00 Å), leading the molecule to adopt a bridge geometry. Although the degree of activation in this mode is significant, it is lower than that observed for IIIA.

Regarding mode IIIC, CO_2_ adsorbs at a hollow site with the carbon atom near Ni (1.91 Å), but without interacting with the Fe adatom. This specific arrangement leads to C–O bond activation to 1.30 Å, primarily driven by the interaction between the oxygen atoms and surface Fe atoms (~1.98 Å) (Figure S3).

A distinctive flat-lying orientation characterizes mode IIID, where the molecule occupies a hollow site and interacts simultaneously with the Fe adatom (C–Fe_ad_ = 2.02 Å) and the Ni atom (O–Ni = 2.19 Å). Although both C–O bonds are strongly activated (1.32–1.33 Å), this configuration exhibits low stability, possibly due to the fact that the oxygen atoms also interact with surface Fe atoms (2.04–2.09 Å). The IIIE configuration involves CO_2_ adopting a bridge geometry between the Fe adatom and a surface Fe site. While still activated, it shows the weakest activation among the IIIA–IIIE series (Table 4), with the Ni atom remaining as a spectator in this adsorption process (Figure S3).

On the Fe_ad_-Ni_1_-Fe(100) surface, charge transfer consistently leads to the formation of negatively charged CO_2_^δ–^ species. However, unlike the previous two systems, the correlation between adsorption stability and charge transfer is not preserved; notably, some less stable configurations (e.g., IIIC and IIID) show the most significant charge transfer. Despite this discrepancy in terms of stability, a consistent trend emerges across all three surfaces when CO_2_ geometric distortion is considered, primarily manifested as C–O bond elongation. The relationship between the charge gained by CO_2_ and its most elongated C–O bond for all adsorption modes is illustrated in Figure S4. This confirms the expected linear correlation between charge transfer and C–O bond elongation across all adsorption modes. This behavior is consistent with previous reports for other catalytic surfaces [25].

Hollow-site configurations exhibit the largest charge accumulation on CO_2_. Specifically, mode IIID displays the highest charge transfer, not only within the Fe_ad_-Ni_1_-Fe(100) surface but also when compared to systems involving substitutional Ni or Ni adatoms on Fe(100). This behavior is attributed to the simultaneous and more extensive stretching of both C–O bonds (1.33 and 1.32 Å).

**Figure 3:**
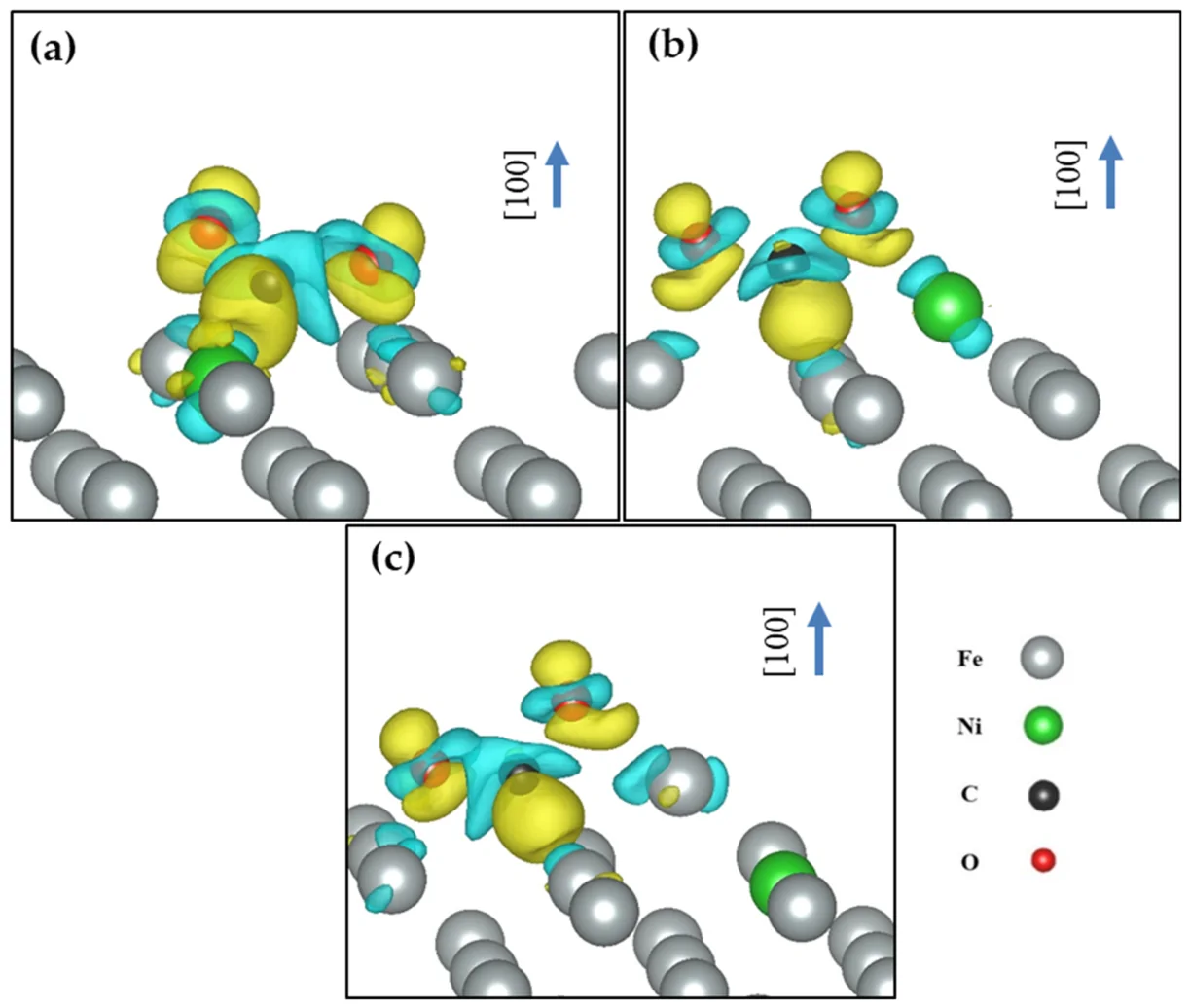
Charge density difference (Δρ) plots for: (**a**) Ni_1_-Fe(100)-IA, (**b**) Ni_ad_-Fe(100)-IIA, and (**c**) Fe_ad_-Ni_1_-Fe(100)-IIIA configurations. The isosurfaces are rendered at Δρ = ± 9 × 10^−3^ Bohr^−3^. Yellow and light blue regions denote electronic charge accumulation and depletion, respectively. Color code: Fe (light grey), Ni (green), C (dark grey), and O (red).

A complementary way to visualize the electronic charge reorganization upon CO_2_ adsorption is through electron charge density difference (Δρ) plots (Figure 3). For the most stable configurations, these maps clearly illustrate the charge redistribution and the transfer of electronic density from the surfaces to the CO_2_ molecule. The predominance of blue regions surrounding the molecule indicates electron accumulation. This visual evidence is consistent with the DDEC6 charge values calculated for CO_2_ in each system, confirming that the molecule becomes negatively charged, as previously discussed (Table 1, Table 3 and Table 4).

In summary, CO_2_ adsorbs on Ni_1_-Fe(100) and Ni_ad_-Fe(100) with slightly lower binding energy than on pure Fe(100) (1.02–1.03 eV) [25]. Consequently, adsorption will predominantly occur on the more abundant Fe(100) surface; however, the bimetallic sites are likely to be occupied as well, as the energy difference is a mere 0.06 eV. On the other hand, CO_2_ chemisorbs on Fe_ad_-Ni_1_-Fe(100) more strongly than on Fe(100); therefore, adsorption is expected to preferentially take place at the IIIA site in this system.

### 3.2. CO_2_ Dissociation on Ni_1_-Fe(100), Ni_ad_-Fe(100), and Fe_ad_-Ni_1_-Fe(100)

While stable adsorption is a key factor, sufficient activation is equally crucial to lower the energy barrier for subsequent C–O bond breaking. In some cases studied, the most stable structures are also the most activated; however, in others, highly activated configurations exhibit significantly lower relative stability. Consequently, both types of configurations were examined to identify those representing a feasible compromise between stability and activation for further dissociation analysis.

**Figure 4:**
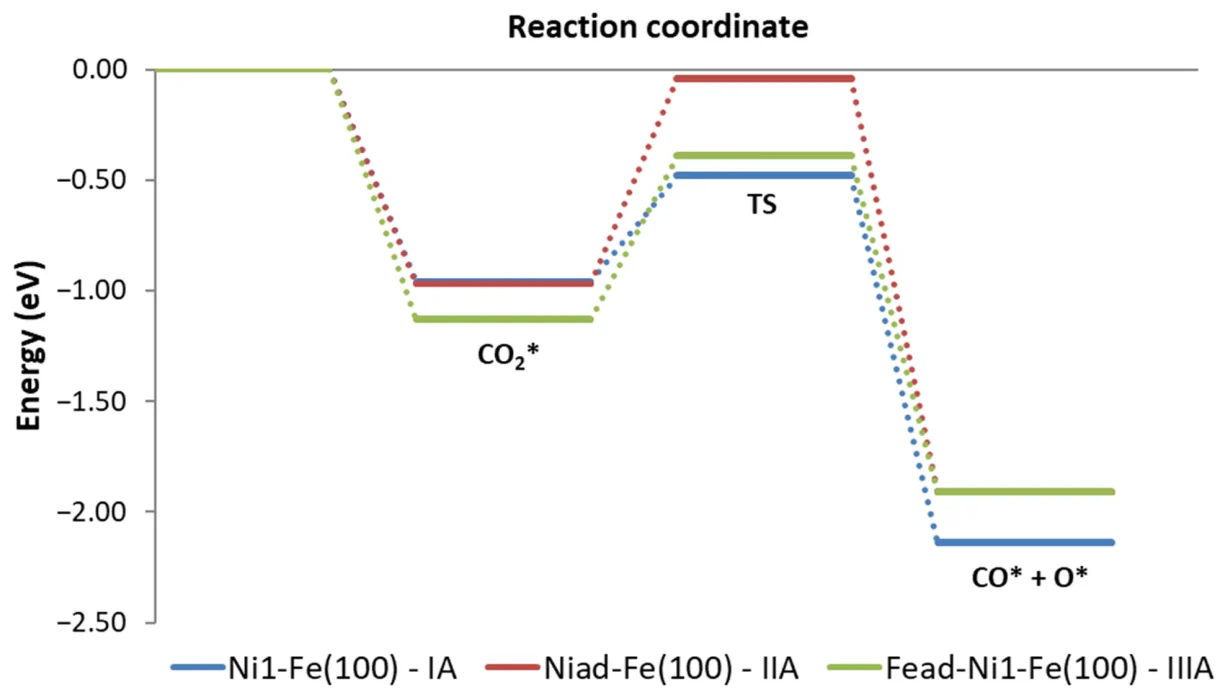
Energy profiles for CO_2_ dissociation on Ni_1_-Fe(100)-IA, Ni_ad_-Fe(100)-IIA and Fe_ad_-Ni_1_-Fe(100)-IIIA. The pure Fe(100) activation barrier (E_act_ = 0.84 eV) is included for comparison [25]. Energies are reported relative to the clean surface plus isolated gas-phase CO_2_. The profile displays initial (CO_2_*), transition (TS), and final (CO* + O*) states; intermediate NEB images are omitted for clarity. Numerical convergence tests indicate an uncertainty on the order of ~0.01 eV for the activation energies [46].

For each surface, we selected the most stable and representative activated configurations of adsorbed CO_2_*, where the asterisk (*) denotes adsorbed species. Several final states associated with CO + O* co-adsorption were analyzed, but only the most stable configurations near the corresponding initial states were considered to ensure energetically favorable reaction pathways. The CO* + O* co-adsorption configurations identified are summarized in Table S5. Figure 2 shows the reaction pathways for CO_2_ dissociation, including the adsorption geometries of the initial (IS), transition (TS), and final (FS) states, while Figure 4 presents the corresponding dissociation energy profiles for the best-performing configurations on each surface.

#### 3.2.1. CO_2_ Dissociation on Ni_1_-Fe(100)

For the Ni_1_-Fe(100) substrate, the IA configuration (E_ads_ = −0.96 eV) is both the most stable and the most activated, exhibiting the lowest CO_2_ dissociation barrier among all systems studied (E_act_ = 0.48 eV; Table 5). Upon dissociation, both CO* and O* fragments occupy hollow sites (Figure 2a). The reaction energy displays an intermediate level of exothermicity relative to the other systems (Table 5).

The dissociation barrier obtained here is significantly lower than those reported by Nie et al. [45] for various Ni coverages on Fe(100) (0.8–1.0 eV). In their specific Ni_1_-Fe(100) case, the active site does not involve interaction with Ni, resulting in a barrier of 0.8 eV. Notably, the IA adsorption geometry is only 0.06 eV less stable than the corresponding mode on Fe(100), while the CO_2_ dissociation barrier is 0.36 eV lower. This value also improves upon the barrier previously obtained by our group for the same Ni-substituted system on Fe(111) (0.88 eV) [19]. Moreover, the barrier is lower than the 0.80 eV calculated by Wang et al. [47] for CO_2_ dissociation on pure Fe(100), while maintaining a similar reaction energy (E_reac_ = −1.19 eV).

The CO_2_ dissociation barrier from the IB site is much higher than from IA and comparable to that on pristine Fe(100) (0.84 eV) [25], making dissociation from this geometry unlikely due to its lower adsorption stability. This configuration, which is less stable than IA, was nevertheless analyzed because it shows a strongly activated C–O bond but presents an opposite arrangement, where C and O interact with Fe and Ni, respectively.

#### 3.2.2. CO_2_ Dissociation on Ni_ad_-Fe(100)

For the Ni_ad_-Fe(100) surface, configuration IIA is the most stable adsorption geometry and also shows the highest degree of activation. Its adsorption energy is comparable to that of IA on the Ni_1_-Fe(100) substrate (Table 1 and Table 3). However, introducing Ni as an adatom significantly increases the dissociation barrier, yielding an E_act_ of 0.93 eV, which is considerably higher than the value found for the substitutional Ni case (IA) (Table 5). Upon dissociation, the CO* fragment remains in a bridge position, whereas O* migrates to a neighboring hollow site (Figure 2b).

Comparing the effect of a Ni adatom on two different crystallographic planes, (100) and (111), we find that the barrier for IIA is 0.16 eV higher. This result indicates that the Ni adatom is less effective for CO_2_ dissociation when deposited on the (100) plane than on the (111) surface (0.86 eV) [19].

#### 3.2.3. CO_2_ Dissociation on Fe_ad_-Ni_1_-Fe(100)

The IIIA configuration exhibits the strongest CO_2_ adsorption among all surfaces studied, as evidenced by its adsorption energy (Table 4). In terms of reaction energies, this system is the least exothermic of the three, yet it shows the second-lowest CO_2_ dissociation barrier, with an E_act_ of 0.74 eV (Table 5). When compared with the value previously obtained for the Fe_ad_-Fe(100) system [25], the difference is only 0.12 eV, with the latter being slightly more favorable. However, when comparing Fe_ad_-Ni_1_-Fe(100) with the Ni_ad_-Fe(100) surface —both of which have the same total number of atoms—the former shows a significantly lower barrier. The activation energy decreases by 0.19 eV when Ni is placed in a substitutional site with Fe occupying the adatom site in this Fe_ad_-Ni_1_-Fe(100) system. Although this surface facilitates CO_2_ dissociation more readily than Ni_ad_-Fe(100) and pure Fe(100) (E_act_ = 0.84 eV) [25], no additional synergistic enhancement beyond the individual effects of substitutional Ni or Fe adatoms was observed, since the barrier is higher than those for Fe_ad_-Fe(100) and Ni_1_-Fe(100).

When the most activated C–O bond (1.34 Å) breaks, the oxygen atom that dissociates initially interacts with a surface Fe atom rather than with the Fe adatom (Figure 2c). In the transition state, the activated C–O bond stretches to 1.77 Å, and the C atom approaches a surface Fe atom before reaching its final position. Upon dissociation, the CO* fragment binds to a surface Fe atom in a slightly tilted bridge configuration (C–Fe = 1.77 Å), while O* occupies a hollow site, its most stable adsorption geometry.

Given the minimal energy difference between the IIIA and IIIB configurations, the dissociation process was also investigated starting from the latter. Although the C–O bonds in IIIB are not as elongated as in IIIA, the activation barrier of 0.77 eV was found to be similar to the value obtained from the IIIA site. Thus, CO_2_ is expected to preferentially adsorb and dissociate from both sites more readily than on Fe(100).

From the data presented in Figure 4 and Table 5, we have highlighted distinct behaviors for each system:

- Ni_1_-Fe(100) exhibits the *most exothermic* reaction energy (E_reac_ = −1.18 eV) and the *lowest* activation barrier (E_act_ = 0.48 eV). Thus, CO_2_ dissociation is both thermodynamically and kinetically highly favorable on this surface.
- Ni_ad_-Fe(100) displays a significantly higher barrier (0.93 eV) and a less exothermic reaction (−0.94 eV). Although CO_2_ adsorption is similar to that of IA (−0.97 eV), the transition state is poorly stabilized (−0.04 eV), suggesting slower kinetics.
- Fe_ad_-Ni_1_-Fe(100) shows an intermediate barrier (0.74 eV) and moderately exothermic reaction energy (−0.78 eV). This configuration yields the strongest CO_2_ adsorption (−1.13 eV), indicating significant stabilization prior to dissociation. However, this strong binding slightly increases the barrier relative to the IA case on Ni_1_-Fe(100).

In summary, the activation barriers follow the order (including the pure Fe(100) surface):

Ni_1_-Fe(100) << Fe_ad_-Ni_1_-Fe(100) < Fe(100) < Ni_ad_-Fe(100)

This trend clearly identifies the Ni_1_-Fe(100) system as the most efficient catalyst for CO_2_ dissociation, combining a low activation barrier with a strongly exothermic reaction. The significant energy difference between the first two bimetallic systems implies a substantial increase in the reaction rate according to the Arrhenius law, potentially corresponding to several orders of magnitude depending on the reaction temperature. Fe_ad_-Ni_1_-Fe(100) provides a balanced compromise between activity and stability, while Ni_ad_-Fe(100) is limited mainly by slower kinetics.

### 3.3. Bond-Order Analysis

Bond-order (BO) analysis was used as a quantitative measure of the electron density shared between two atomic centers, facilitating the identification of the strength of their chemical interactions.

Analysis of the dissociating C–O bond order reveals distinct trends across the surfaces. On Ni_ad_-Fe(100), the BO value at the initial state (IS) is the highest (least activated), while at the transition state (TS) it is the lowest (most elongated), indicating the latest TS geometry (see IS and TS in Table 2 and Table S6, respectively). This difference is smaller on the other surfaces, suggesting greater efficiency in stabilizing the fragment while still bonded. During dissociation, C–O bond order values decrease while the corresponding values of Me–O and Me–C bonds increase, indicating synchronous bond evolution.

The C atom interacts primarily with Ni on the Ni_1_-Fe(100) surface, whereas on the other two surfaces it coordinates with surface Fe atoms (bond order ~0.8). This difference is essential when evaluating magnetic behavior in the next section. Regarding the oxygen atom of the activated C–O bond, the O atom coordinates with two surface Fe atoms on Ni_1_-Fe(100) and Fe_ad_-Ni_1_-Fe(100), while on Ni_ad_-Fe(100) it binds only to Ni but remains relatively far from the surface Fe atoms. This configuration hinders dissociation, as reflected in the highest C–O bond order among the three systems (Table 2).

Intermetallic bond order variations between IS and TS are minor. However, the Ni–Fe interaction strength increases when the adatom is Fe, as on the Fe_ad_-Ni_1_-Fe(100) surface. This Fe_ad_-Ni interaction is also stronger than on the equivalent monometallic surface (Fe_ad_–Fe = 0.49), disfavoring C coordination with Fe_ad_ at the TS—opposite to the behavior observed on the Fe_ad_-Fe(100) surface (0.23 vs. 0.39 on Fe_ad_-Ni_1_-Fe(100) and Fe_ad_-Fe(100), respectively) [25]. Likely, this reduced C-Fe_ad_ coordination destabilizes the activated complex on the bimetallic surface, increasing the C–O dissociation barrier by 0.12 eV compared to the equivalent monometallic surface.

In summary, C-Ni and O-Fe interactions are both favorable for C–O bond cleavage, regardless of whether Fe is part of the surface or an adatom.

### 3.4. Electronic Structure, LDOS, and d-Band Analysis

To deepen our understanding of the activation barriers, we examined the local density of states (LDOS) and computed the corresponding d-band centers (E_d_) for pristine surfaces, as well as for the IS with adsorbed CO_2_, and the TS configurations. According to the Hammer–Nørskov model [48,49], the position of the d-band center relative to the Fermi level (E_F_) governs adsorbate–metal interactions: a d-band center closer to E_F_ corresponds to greater orbital overlap, enhanced hybridization, and stronger binding (Figure S5). Here, we consider the overall d-band center (spin-averaged).

For Ni_1_–Fe(100), we analyzed the Ni atom and a neighboring Fe atom. The d-band center of the Fe atom is −0.92 eV (Table 6), compared to −1.02 eV for pure Fe(100) [25]. This slight upward shift suggests a ligand effect: the substitutional Ni dopant modifies the electronic environment of neighboring Fe atoms, which may enhance CO_2_ hybridization and reactivity. Such ligand effects are well documented in bimetallic catalysts [50,51,52].

In the case of Ni_ad_-Fe(100), an Fe atom—bonded to the Ni adatom and C atom—shows only a slight shift (−1.02 → −0.99 eV), indicating a weak ligand effect, as the Ni adatom perturbs the surface less than substitutional Ni. For Fe_ad_-Ni_1_-Fe(100), the Fe d-band center reaches −0.75 eV, the highest among the three surfaces. This Fe atom experiences dual perturbation from close interactions with both the Ni atom and Fe adatom. Based on d-band theory alone, this surface should be the most reactive; however, it exhibits only an intermediate barrier (0.74 eV), motivating a deeper analysis.

### 3.5. Role of Spin Polarization

In magnetic metals such as Fe, Co, and Ni, it is appropriate to analyze the spin-up (↑) and spin-down (↓) channels separately because the average d-band model is inadequate to capture the true catalytic reactivity [53]. Magnetization causes the d-band centers of each channel to shift in opposite directions relative to the Fermi level, creating an electronic asymmetry. Furthermore, the Stoner criterion defines whether a metal will be ferromagnetic based on the competition between kinetic energy and exchange energy (spin alignment) [53]. In catalytic processes, strong chemisorption induces redistribution of numerous electronic states from near E_F_ toward lower energies due to hybridization and bond formation with the adsorbate. The result is the local perturbation of the Stoner criterion, and the energy penalty depends on the magnetic rigidity of the metal when reconfiguring spin channels to stabilize the new chemical bond. This phenomenon explains why adsorption on magnetic systems is energetically less favorable than on non-magnetic ones [53,54]. So, in magnetic transition-metal surfaces, adsorption not only involves charge redistribution and orbital hybridization, but also spin reorganization effects associated with the local perturbation of the Stoner exchange splitting. Consequently, part of the adsorption energy is associated with the energetic cost of reconfiguring the spin channels and local magnetic moments to stabilize the newly formed chemical bond. Therefore, the catalytic activity cannot always be rationalized solely through the average d-band center, since the spin-dependent electronic structure may introduce additional stabilization or energetic penalties during bond activation.

Spin polarization of the metal d-states was examined by performing a detailed analysis of the d-band centers relative to the Fermi level (E_F_) for the spin-up (E_↑_) and spin-down (E_↓_) channels, including the spin splitting (ΔE). Accordingly, we computed spin-resolved LDOS for the Fe and Ni atoms bonded to CO_2_ in each system (Figure S6).

For Ni_1_-Fe(100), ΔE of the Fe atom remains robust and nearly stable, shifting only from −2.89 eV (clean surface) to −2.71 eV (TS) (Table 7). However, strong CO_2_ adsorption through the C atom on Ni causes a downshift of the spin-down d-band channel (stabilization effect), reducing the spin splitting by 30–40%. This channel approach indicates that the metal locally reorganizes its magnetism with high efficiency and low energetic cost, owing to the greater magnetic flexibility of Ni compared to Fe. Consequently, this system exhibits the lowest energy cost for CO_2_ dissociation (0.48 eV).

In contrast, for Ni_ad_-Fe(100), the spin splitting (ΔE = E_↑_ − E_↓_) of the Fe atom interacting with the C atom decreases from −2.83 eV on the clean surface to −2.09 eV at the transition state, due to an upshift of the spin-up d-band channel toward energies closer to the Fermi level (destabilization effect). This significant reduction in the exchange spin splitting (by 0.74 eV) manifests as a substantial decrease in the Fe atom spin moment, from 2.89 to 2.09 μ*_B_*. Additionally, the Ni spin splitting decreases. These magnetization changes penalize the C–O bond-breaking stage, substantially increasing the CO_2_ dissociation barrier (0.93 eV) for this surface compared to the substitutional Ni case.

For Fe_ad_-Ni_1_-Fe(100), the situation is intermediate between the previous two surfaces: reductions in Fe spin splitting are less pronounced, requiring less reorganization to dissociate CO_2_, consistent with its moderate activation energy. Although the overall Fe d-band center (E_↑_ + E_↓_) is the highest (−0.75 eV), the spin-resolved centers shift significantly from −2.60 eV (clean surface) to −2.26 eV (TS). This substantial magnetic and electronic reorganization likely penalizes activation, contributing to the intermediate barrier.

These results align with studies highlighting the importance of spin-resolved d-band structure—not merely the average d-band center—in CO_2_ and similar catalytic reactions [53,54]. The system must expend energy to overcome local magnetic stability (governed by the Stoner criterion) for effective electronic hybridization with the adsorbate. Thus, the lowest activation barrier is associated with the surface where demagnetization occurs most efficiently at minimal energetic cost.

Finally our results demonstrate that a substitutional Ni dopant (integrated into the lattice) performs better than adatom configurations for CO_2_ dissociation on these surfaces. While the substitutional site maintains a favorable electronic environment, its primary advantage lies in enabling a more efficient local spin reorganization during bond dissociation. In contrast, adatom structures promote stronger electronic localization and enhanced magnetic rigidity, which introduce an additional kinetic penalty along the reaction pathway.

It is important to note that CO_2_ dissociation activity alone does not guarantee good overall catalytic performance, since the fate of the resulting C and O adatoms plays a critical role in the actual efficiency of the catalyst. In this work, we focused on the initial activation and dissociation of CO_2_, a key first step in several catalytic processes involving CO_2_ as a reactant. Further steps, including diffusion, recombination, and possible surface poisoning by C and O species, as well as their removal through reaction with co-adsorbed species, remain to be addressed in future work.

Despite these open questions, and although directly comparable experimental data for this system remain scarce, the results obtained here offer valuable guidance for future experimental efforts. In particular, the notably low CO_2_ dissociation barrier found on Ni1-Fe(100) suggests that this surface is a promising candidate for reactions in which CO_2_ is used as a feedstock. These findings provide a predictive framework that can help experimentalists prioritize systems and conditions for further catalytic evaluation.

## 4. Conclusions

In this work, we evaluated CO_2_ adsorption and dissociation on three bimetallic Ni-Fe surfaces. The systems were characterized by analyzing geometries using the Bond Order analysis, along with energetic properties, charge density distributions, and local density of states (LDOS).

On each surface, several activated CO_2_ adsorption geometries were identified, as confirmed by electron transfer from the surface to the molecule, forming CO_2_^δ–^ species. Among these, we selected the most stable geometries exhibiting the highest degree of activation, for which the CO_2_ dissociation barriers follow the order: Ni_1_-Fe(100) << Fe_ad_-Ni_1_-Fe(100) < Ni_ad_-Fe(100).

Ni_1_-Fe(100) was identified as the optimal surface, displaying the most exothermic reaction energy and the lowest activation barrier, indicating that CO_2_ dissociation is both thermodynamically and kinetically favored. The substitutional Ni introduces a ligand effect on the Fe(100) surface that modifies the electronic structure of neighboring Fe atoms through charge transfer, d-band center shifts, and changes in spin polarization. These effects alter the interaction with the CO_2_ molecular orbitals and promote C–O bond cleavage relative to pure Fe, making Ni_1_-Fe(100) the most favorable system.

The detailed analysis of spin-resolved LDOS for the corresponding Fe and Ni atoms acting as active sites was fundamental to interpreting these results. The simple d-band center model alone is insufficient to explain the catalytic activity of these magnetic systems.

From a synthesis perspective, our results suggest that experimental efforts should prioritize methods capable of producing substitutional Ni rather than isolated Ni adatoms, given the distinct catalytic behavior observed between these two configurations. These findings can help guide the rational design of Ni-Fe catalysts with tailored active-site configurations for CO_2_ activation.

## Figures and Tables

**Table 1:** Adsorption energies (E_ads_, ZPE-corrected), C-O bond distances (d), and DDEC6 CO_2_ charges (*q*) for CO_2_ adsorption on Ni_1_-Fe(100).

Ni_1_-Fe(100)	E_ads_ (eV)	d(C-O) (Å)	*q* CO_2_ (*e*)
IA	−0.96	1.35	−0.32
IB	−0.77	1.34	−0.32
IC	−0.74	1.34	−0.33
ID	−0.49	1.30	−0.29

**Table 2:** Relevant interatomic distances (in Å) and bond orders (in parentheses) ^†^ for the most stable CO_2_ adsorption configurations on Ni_1_-Fe(100), Ni_ad_-Fe(100), and Fe_ad_-Ni_1_-Fe(100) surfaces. D* denotes the most activated C-O bond length in CO_2_.

	Ni_1_-Fe(100)—IA	Ni_ad_-Fe(100)—IIA	Fe_ad_-Ni_1_-Fe(100)—IIIA
D* C-O_A_^#^	1.35 (1.39)	1.29 (1.67)	1.34 (1.46)
d C-Ni	1.92 (0.78)	---	---
d C-Fe_sup_^‡^	2.24 (0.25)	1.94 (0.81)	1.96 (0.75)
d C-Fe_ad_	---	---	2.37 (0.15)
d O_A_-Ni	---	1.94 (0.57)	---
d O_A_-Fe_sup_	2.03 (0.49) 2.10 (0.41)	3.43 (0.02)	2.05 (0.48) 2.14 (0.36)
d O-Fe_sup_	2.00 (0.54)	2.00 (0.57)	---
d O-Fe_ad_	---	---	2.00 (0.53)
d Ni-Fe_sup_	2.85 (0.18)	---	2.74 (0.24) 2.92 (0.14)
d Ni_ad_-Fe_sup_	---	2.40 (0.38)	---
d Fe_sup_-Fe_ad_	---	---	2.36 (0.49)
d Ni-Fe_ad_	---	---	2.35 (0.59)

^†^ The C-O bond order in gas-phase CO_2_ is 2.08 (DDEC6 value calculated). ^#^ O_A_ = oxygen atom of the most activated C-O bond. ^‡^ Fe_sup_ = Fe superficial.

**Table 3:** Adsorption energies (E_ads_, ZPE-corrected), C-O bond distances (d), and DDEC6 CO_2_ charges (*q*) for CO_2_ adsorption on Ni_ad_-Fe(100).

Ni_ad_-Fe(100)	E_ads_ (eV)	d(C-O) (Å)	*q* CO_2_ (*e*)
IIA	−0.97	1.29	−0.29
IIB	−0.44	1.26	−0.20
IIC	−0.23	1.26	−0.18

**Table 4:** Adsorption energies (E_ads_, ZPE-corrected), C-O bond distances (d), and DDEC6 CO_2_ charges (*q*) for CO_2_ adsorption on Fe_ad_-Ni_1_-Fe(100).

Fe_ad_-Ni_1_-Fe(100)	E_ads_ (eV)	d(C-O) (Å)	*q* CO_2_ (*e*)
IIIA	−1.13	1.34	−0.29
IIIB	−1.07	1.29	−0.26
IIIC	−0.89	1.30	−0.33
IIID	−0.75	1.33	−0.35
IIIE	−0.73	1.26	−0.19

**Table 5:** Adsorption energies (E_ads_), reaction energies (E_reac_) and activation barriers (E_act_), in eV, for CO_2_ dissociation on Ni_1_-Fe(100), Ni_ad_-Fe(100), and Fe_ad_-Ni_1_-Fe(100) are summarized. All energy values include ZPE corrections.

Surface CO_2_* → CO*+ O* Reaction †	E_ads_	E_reac_	E_act_
**Ni** **_1_** **-Fe(100)**			
IA	−0.96	−1.18	0.48
IB	−0.77	−0.95	0.81
**Ni_ad_-Fe(100)**			
IIA	−0.97	−0.94	0.93
**Fe_ad_-Ni** **_1_** **-Fe(100)**			
IIIA	−1.13	−0.78	0.74
IIIB	−1.07	−0.69	0.78

† E_act_ = 0.84 eV on Fe(100) [25].

**Table 6:** d-band center (E_d_) of a superficial Fe atom for clean surfaces, and for IS and TS on each most stable system of surfaces Ni_1_-Fe(100)-IA, Ni_ad_-Fe(100)-IIA and Fe_ad_-Ni_1_-Fe(100)-IIIA.

E_d_ (eV)	Clean Surface	IS	TS
Ni_1_-Fe(100)	−0.92	−0.84	−0.76
Ni_ad_-Fe(100)	−0.99	−0.88	−0.80
Fe_ad_-Ni_1_-Fe(100)	−0.75	−0.70	−0.69

**Table 7:** d-band center values of spin-up (↑) and spin-down (↓) for Fe and Ni atoms interacting with CO_2_ of the most stable systems on each surface, and the spin splitting (∆E) (both expressed in eV).

	Clean Surface		IS		TS	
	Ni	Fe	Ni	Fe	Ni	Fe
**Ni_1_-Fe(100)**						
**E_d_**	−1.20	−0.92	−1.37	−0.84	−1.35	−0.76
**E_↑_** **_/_** **E_↓_**	−1.62/−0.80	−2.35/0.54	−1.63/−1.14	−2.21/0.47	−1.63/−1.07	−2.14/0.57
**∆E**	−0.82	−2.89	−0.49	−2.68	−0.56	−2.71
**Link with:**	---	---	C	O	C	O
**Ni_ad_-Fe(100)**						
**E_d_**	−1.57	−0.99	−1.59	−0.88	−1.56	−0.80
**E_↑_** **_/_** **E_↓_**	−2.12/−1.05	−2.42/0.41	−2.01/−1.16	−1.85/0.16	−1.93/−1.20	−1.85/0.24
**∆E**	−1.07	−2.83	−0.85	−2.01	−0.73	−2.09
**Link with:**	---	---	O	C	O	C
**Fe_ad_-Ni_1_-Fe(100)**						
**E_d_**	−1.45	−0.75	−1.38	−0.70	−1.41	−0.69
**E_↑_** **_/_** **E_↓_**	−1.89/−1.06	−2.08/0.52	−1.85/−0.88	−1.75/0.38	−1.86/−0.95	−1.79/0.47
**∆E**	−0.83	−2.60	−0.97	−2.13	−0.91	−2.26
**Link with:**	---	---	---	C	---	C

## Data Availability

The original contributions presented in this study are included in the article/Supplementary Materials. Further inquiries can be directed to the corresponding authors.

## Supplementary Materials

The following supporting information can be downloaded at: https://www.mdpi.com/article/10.3390/nano16181165/s1, Figure S1: Adsorption geometries for CO_2_ adsorptions on Ni_1_-Fe(100). Color code: Fe (light grey), Ni (green), C (dark grey), and O (red).; Figure S2: Adsorption geometries for CO_2_ adsorptions on Ni_ad_-Fe(100). Color code: Fe (light grey), Ni (green), C (dark grey), and O (red); Figure S3: Adsorption geometries for CO_2_ adsorptions on Fe_ad_-Ni_1_-Fe(100). Color code: Fe (light grey), Fe adatom (orange), Ni (green), C (dark grey), and O (red); Figure S4: Relationship between the charge gained by CO_2_ and the most elongated C–O bond, for all adsorption modes on the three Ni-Fe surfaces. The lineal fit with the corresponding parameters is shown as an inset in the graph; Figure S5: Local density of states (LDOS) for the clean (a) Ni_1_-Fe(100), (b) Ni_ad_-Fe(100), and (c) Fe_ad_-Ni_1_-Fe(100). The vertical line at zero energy denotes the Fermi level; Figure S6: Local density of states (LDOS) for initial states (IS) and transition states of CO_2_ dissociation (TS) on (a) Ni_1_-Fe(100)-IA, (b) Ni_ad_-Fe(100)-IIA, and (c) Fe_ad_-Ni_1_-Fe(100)-IIIA. The vertical line at zero energy denotes the Fermi level; Table S1: Adsorption energies (E_ads_) with and without ZPE correction, for CO_2_ adsorption on Ni_1_-Fe(100); Table S2: Adsorption energies (E_ads_) with and without ZPE correction, for CO_2_ adsorption on Ni_ad_-Fe(100); Table S3: Adsorption energies (E_ads_) with and without ZPE correction, for CO_2_ adsorption on Fe_ad_-Ni_1_-Fe(100); Table S4: Adsorption energies (E_ads_), reaction energies (E_reac_) and activation barriers (E_act_), in eV, with and without ZPE correction, for CO_2_ dissociation on Ni_1_-Fe(100), Ni_ad_-Fe(100), and Fe_ad_-Ni_1_-Fe(100) are summarized; Table S5: Total energy differences (∆E) for the different CO* + O* co-adsorption configurations on Ni_1_-Fe(100), Ni_ad_-Fe(100), and Fe_ad_-Ni_1_-Fe(100), relative to the co-adsorption configuration selected as the final state for NEB calculations. The adsorption sites indicated for CO* and O* correspond to the final optimized geometries. The subscripts t, b, and h denote top, bridge, and hollow sites, respectively, while the following numbers (e.g., h1, h2, …) distinguish among the different hollow and bridge sites on each surface; Table S6: Minimum CO_2_-substrate interatomic distances (in Å) and corresponding bond order values (in parentheses) for the transition states on Ni_1_-Fe(100), Ni_ad_-Fe(100), and Fe_ad_-Ni_1_-Fe(100) surfaces. D* denotes the most activated C–O bond length in CO_2_.
