# Supplementary material for: Ni-Doping as a Design Strategy for Fe-Based Catalysts: Towards Efficient CO2 Activation and Dissociation Studied by Density Functional Theory
Source: Nanomaterials (Basel). 2026 Sep 16;16(18):1165. doi: 10.3390/nano16181165 (PMC13610678; doi:10.3390/nano16181165)
Supplement: Supplementary file 1 [file nanomaterials-16-01165-s001.zip › nanomaterials-4515634-supplementary.pdf]

Supplementary information:

Ni-Doping as a Design Strategy for Fe-Based Catalysts: Towards Efficient CO_2 Activation and Dissociation Studied by Density Functional Theory

Ana Cecilia Rossi Fernández [1], Silvia Andrea Fuente [1], Ricardo Mario Ferullo [2], Alfredo Juan [1,*] and Patricia Gabriela Belelli [1,*]

[1] Instituto de Física del Sur (IFISUR), Universidad Nacional del Sur (UNS), CONICET, Av. L.N. Alem 1253, Bahía Blanca B8000CPB, Argentina; anarossi@ifisur-conicet.gob.ar (A.C.R.F.); silviafuente@frbb.utn.edu.ar (S.A.F.)

[2] Instituto de Química del Sur (INQUISUR), Universidad Nacional del Sur (UNS), CONICET, Av. L.N. Alem 1253, Bahía Blanca B8000CPB, Argentina; caferull@criba.edu.ar

* Correspondence: cajuan@uns.edu.ar (A.J.); patricia.belelli@uns.edu.ar (P.G.B.)

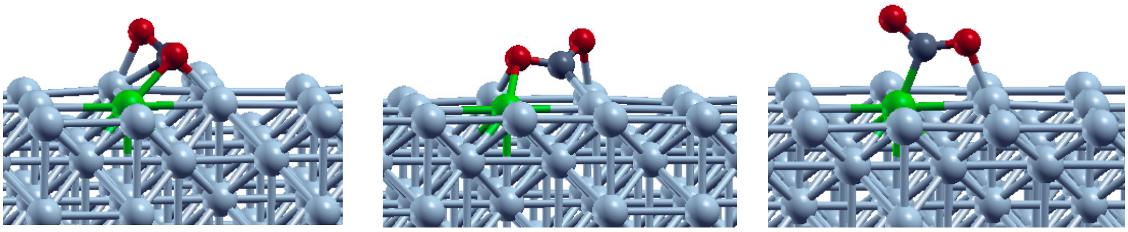

IB IC ID

Figure S1. Adsorption geometries for CO_2 adsorptions on Ni_1-Fe(100). Color code: Fe (light grey), Ni (green), C (dark grey), and O (red).

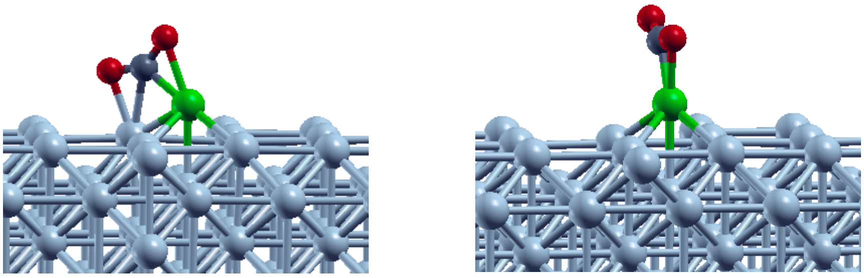

IIB IIC

Figure S2. Adsorption geometries for CO_2 adsorptions on Ni_{ad}-Fe(100). Color code: Fe (light grey), Ni (green), C (dark grey), and O (red).

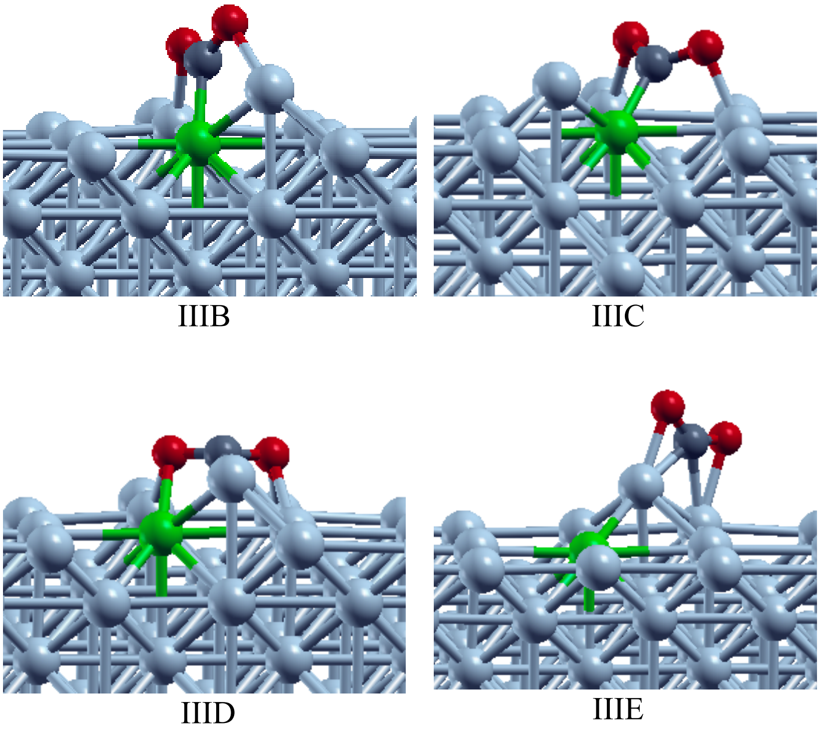

Figure S3. Adsorption geometries for CO_2 adsorptions on Fe_{ad}-Ni_1-Fe(100). Color code: Fe (light grey), Fe adatom (orange), Ni (green), C (dark grey), and O (red).

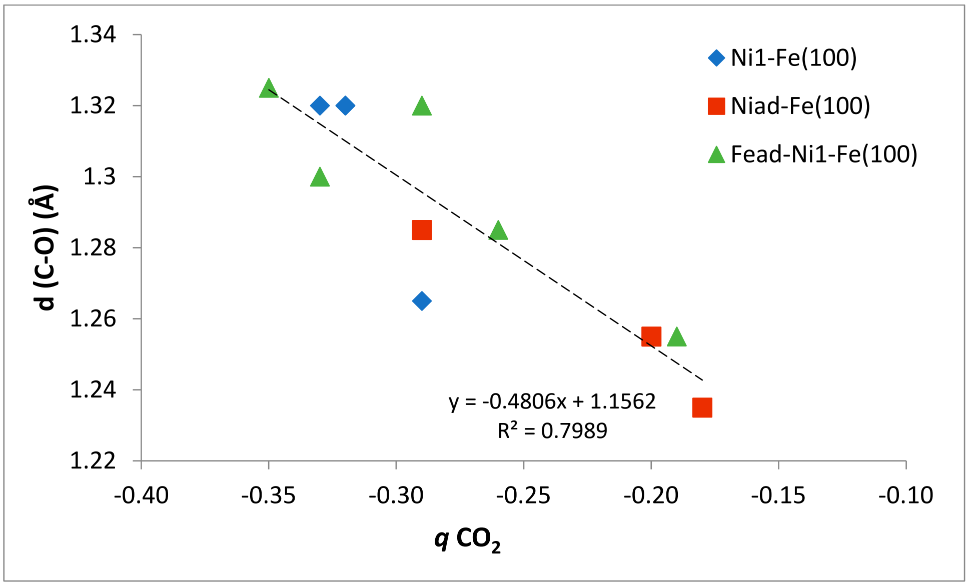

Figure S4. Relationship between the charge gained by CO_2 and the most elongated C–O bond, for all adsorption modes on the three Ni-Fe surfaces. The lineal fit with the corresponding parameters is shown as an inset in the graph.

a)

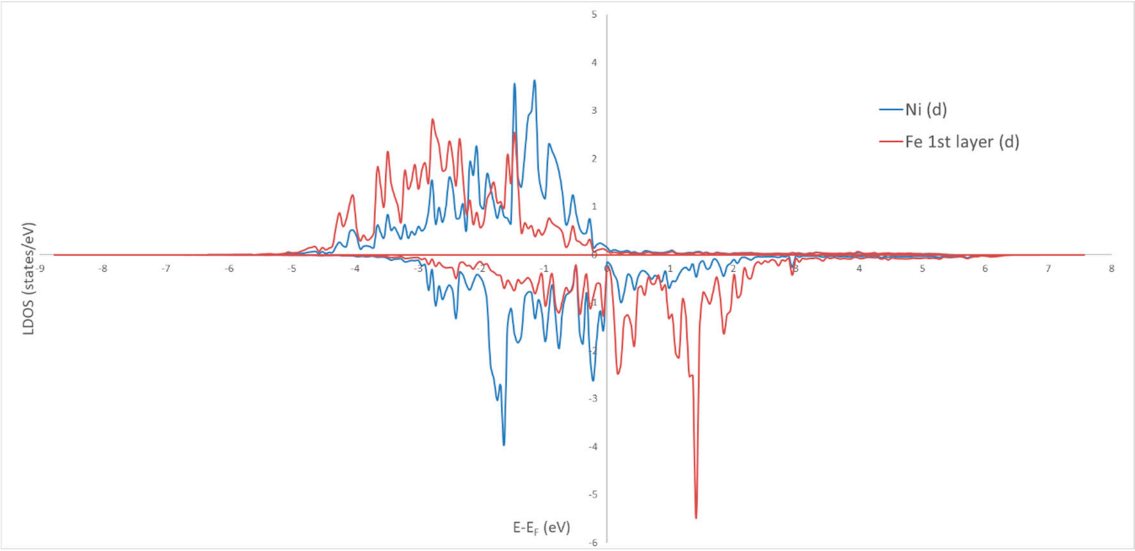

b)

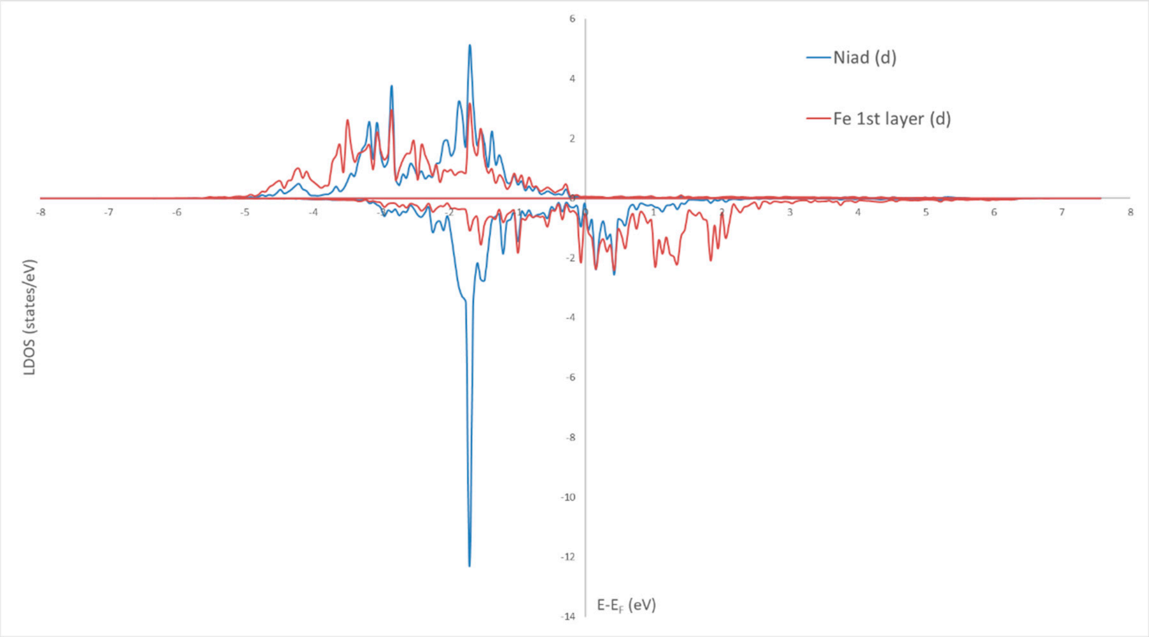

c)

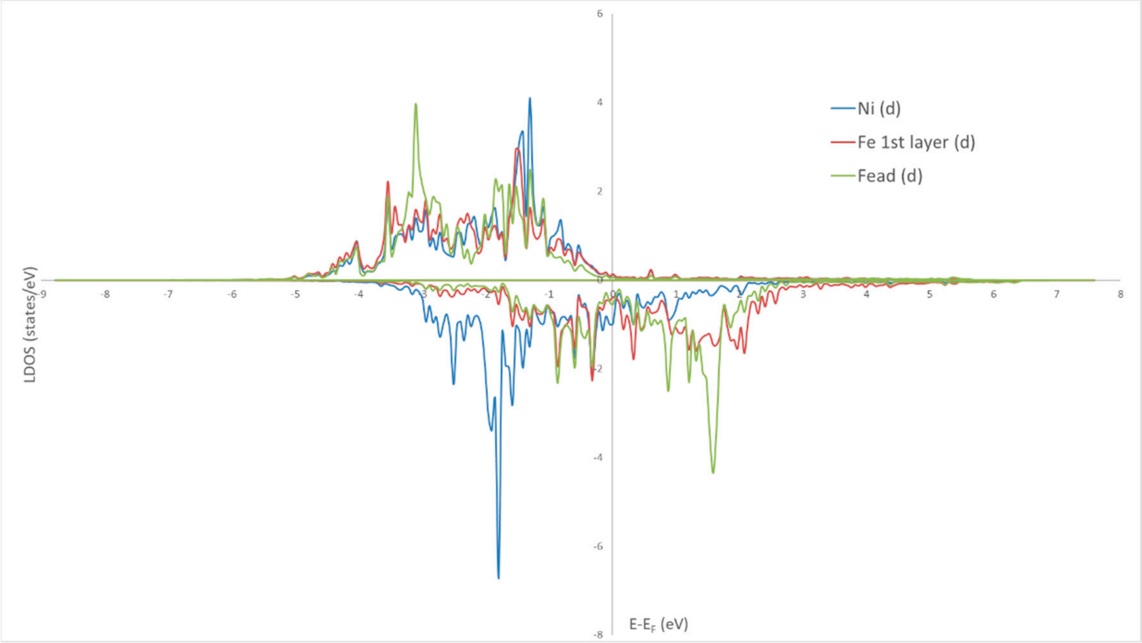

Figure S5. Local density of states (LDOS) for the clean (a) Ni_1-Fe(100), (b) Ni_{ad}-Fe(100), and (c) Fe_{ad}-Ni_1-Fe(100). The vertical line at zero energy denotes the Fermi level.

a)

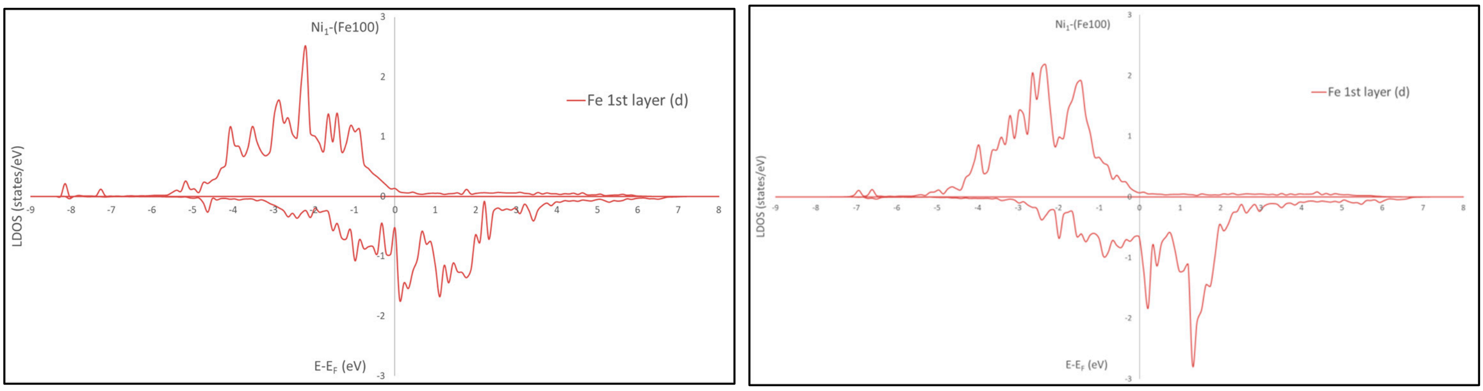

IS **TS**

b)

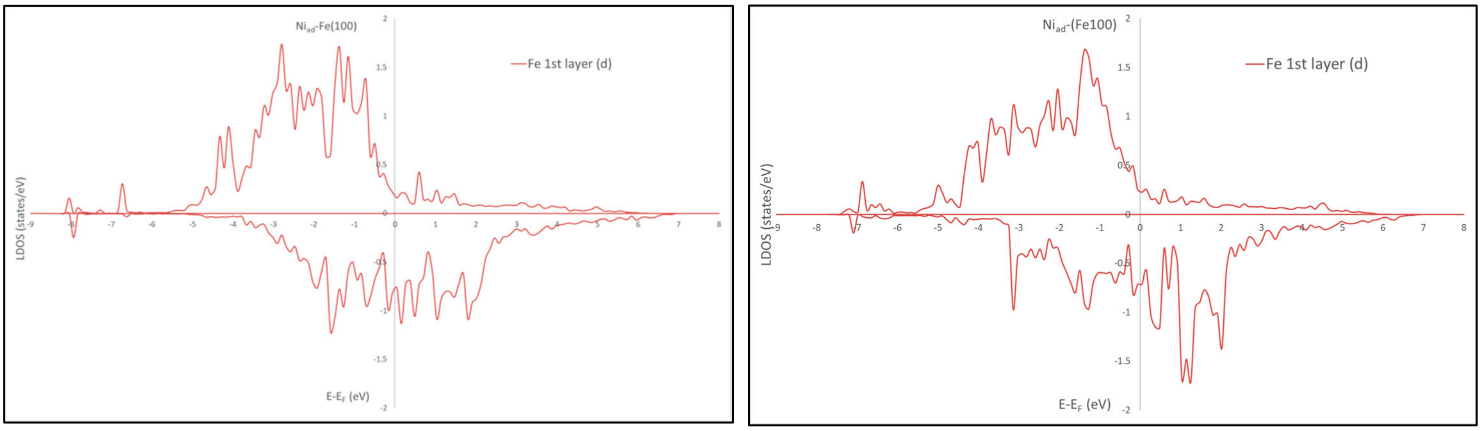

IS **TS**

c)

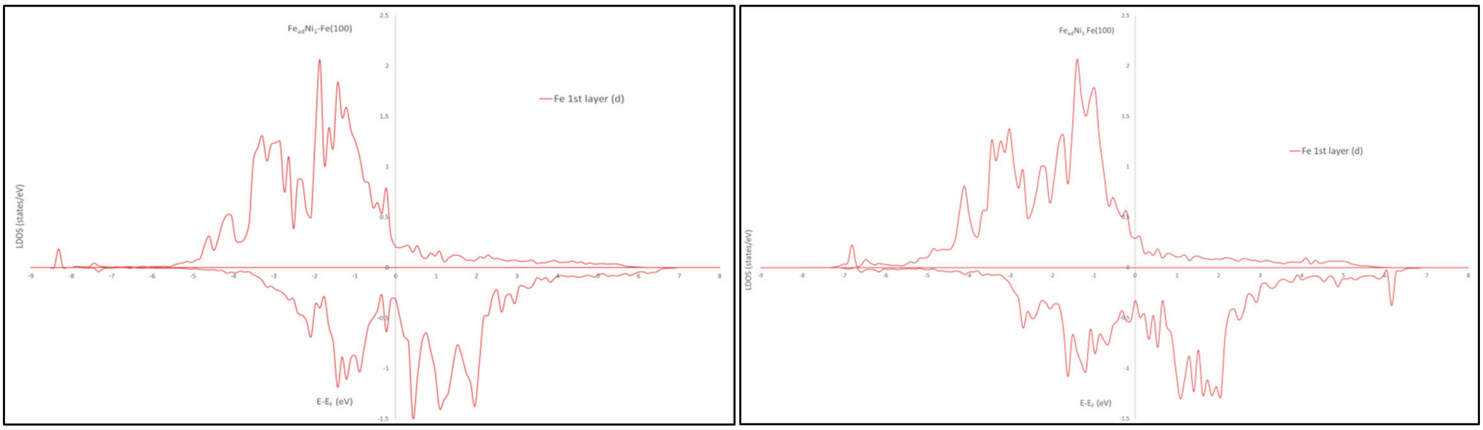

IS **TS**

Figure S6. Local density of states (LDOS) for initial states (IS) and transition states of CO_2 dissociation (TS) on (a) Ni_1-Fe(100)-IA, (b) Ni_{ad}-Fe(100)-IIA, and (c) Fe_{ad}-Ni_1-Fe(100)-IIIA. The vertical line at zero energy denotes the Fermi level.

Table S1. Adsorption energies (E_{ads}) with and without ZPE correction, for CO_2 adsorption on Ni_1-Fe(100).

Ni_1-Fe(100)	E_{ads} (eV) (ZPE)	E_{ads} (eV)
IA	-0.96	-0.93
IB	-0.77	-0.73
IC	-0.74	-0.71
ID	-0.49	-0.48

Table S2. Adsorption energies (E_{ads}) with and without ZPE correction, for CO_2 adsorption on Ni_{ad}-Fe(100).

Ni_{ad}-Fe(100)	E_{ads} (eV) (ZPE)	E_{ads} (eV)
IIA	-0.97	-0.96
IIB	-0.44	-0.41
IIC	-0.23	-0.21

Table S3. Adsorption energies (E_{ads}) with and without ZPE correction, for CO_2 adsorption on Fe_{ad}-Ni_1-Fe(100).

Fe_{ad}-Ni_1-Fe(100)	E_{ads} (eV) (ZPE)	E_{ads} (eV)
IIIA	-1.13	-1.10
IIIB	-1.07	-1.06
IIIC	-0.89	-0.88
IIID	-0.75	-0.72
IIIE	-0.73	-0.71

Table S4. Adsorption energies (E_{ads}), reaction energies (E_{reac}) and activation barriers (E_{act}), in eV, with and without ZPE correction, for CO_2 dissociation on Ni_1-Fe(100), Ni_{ad}-Fe(100), and Fe_{ad}-Ni_1-Fe(100) are summarized.

Surface<br>$CO_2^* \rightarrow CO^* + O^*$ reaction†	E_{ads} (ZPE)	E_{ads}	E_{reac} (ZPE)	E_{reac}	E_{act} (ZPE)	E_{act}
**Ni_1-Fe(100)**						
IA	-0.96	-0.93	-1.18	-1.13	0.48	0.53
IB	-0.77	-0.73	-0.95	-0.90	0.81	0.85
**Ni_{ad}-Fe(100)**						
IIA	-0.97	-0.96	-0.94	-0.89	0.93	1.00
**Fe_{ad}-Ni_1-Fe(100)**						
IIIA	-1.13	-1.10	-0.78	-0.74	0.74	0.79
IIIB	-1.07	-1.06	-0.69	-0.63	0.78	0.86

† $E_{act\text{-}ZPE}$ = 0.84 eV on Fe(100) [23].

Table S5. Total energy differences (ΔE) for the different CO* + O* co-adsorption configurations on Ni_1-Fe(100), Ni_{ad}-Fe(100), and Fe_{ad}-Ni_1-Fe(100), relative to the co-adsorption configuration selected as the final state for NEB calculations. The adsorption sites indicated for CO* and O* correspond to the final optimized geometries. The subscripts t, b, and h denote top, bridge, and hollow sites, respectively, while the following numbers (e.g., h1, h2, …) distinguish among the different hollow and bridge sites on each surface.

CO* + O*/Surface	ΔE (eV)
**CO + O/Ni_1-Fe(100)**	
CO_h* + O_{h1}* (from IA)	0.00
CO_h* + O_{h2}*	0.25
CO_h* + O_{h3}*	-0.19
**CO+O/Ni_{ad}-Fe(100)**	
CO_b* + O_{h1}* (from IIA)	0.00
CO_b* + O_{h2}*	0.40
CO_b* + O_{b1}*	1.00
CO_b* + O_{b2}*	1.00
CO_b* + O_t*	1.00
**CO+O/$Fe_{ad}Ni_1$-Fe100**	
CO_b* + O_{h1}* (from IIIA)	0.00
CO_t* + O_{b2}*	0.21
CO_h* + O_{h2}*	0.03
CO_h* + O_{b1}*	-0.04
CO_h* + O_{h3}*	0.21
CO_h* + O_t*	1.07

Different CO* + O* co-adsorption configurations were considered, and the corresponding optimized energies are summarized in Table S5 (see the caption for the site-labeling convention). It should be noted that the adsorption sites reported in the table correspond to the final optimized geometries and may therefore differ from the initial sites used to construct the configurations. On the Ni_1-Fe(100) surface, one of the co-adsorption configurations was found to be slightly more favorable in energy than the configuration subsequently selected as the final state for the NEB calculation. Nevertheless, this configuration was not considered for the NEB analysis because the O* species is located considerably farther from CO*. Since O* is initially formed at a nearby hollow site upon CO_2 dissociation, it is plausible that it may subsequently migrate toward the more distant hollow site during the reaction.

Table S6. Minimum CO_2-substrate interatomic distances (in Å) and corresponding bond order values (in parentheses) for the transition states on Ni_1-Fe(100), Ni_{ad}-Fe(100), and Fe_{ad}-Ni_1-Fe(100) surfaces. D* denotes the most activated C–O bond length in CO_2.

	**Ni_1-Fe(100) - IA**	**Ni_{ad}-Fe(100) - IIA**	**Fe_{ad}-Ni_1-Fe(100) - IIIA**
D* C-O_A	1.79 (0.45)	1.88 (0.38)	1.77 (0.48)
d C-Ni	1.89 (0.87)	---	---
d C-Fe_{sup}	2.20 (0.37)	1.88 (0.97)	1.95 (0.83)
d C-Fe_{ad}	---	---	2.35 (0.23)
d O_A-Ni	---	1.89 (0.64)	---
d O_A-Fe_{sup}	1.92 (0.70)<br>1.89 (0.78)	1.88 (0.80)	1.87 (0.83)<br>1.94 (0.66)
d O-Fe_{sup}	2.72 (0.09)	2.41 (0.20)	---
d O-Fe_{ad}	---	---	2.88 (0.06)
d Ni-Fe_{sup}	2.78 (0.20)	---	2.71 (0.26)<br>2.93 (0.14)
d Ni_{ad}-Fe_{sup}	---	2.43 (0.38)	---
d Fe_{sup}-Fe_{ad}	---	---	2.35 (0.52)
d Ni-Fe_{ad}	---	---	2.33 (0.62)

O_A = oxygen atom of the most activated C-O bond

Fe_{sup}= Fe superficial
